# The Role of Interleukin-6 and Inflammatory Cytokines in Pancreatic Cancer-Associated Depression

**DOI:** 10.7759/cureus.9969

**Published:** 2020-08-23

**Authors:** Maria Daniela Jarrin Jara, Avneesh S Gautam, Venkata Sri Ramani Peesapati, Mohammad Sadik, Safeera Khan

**Affiliations:** 1 Internal Medicine, California Institute of Behavorial Neurosciences and Psychology, Fairfield, USA; 2 Medicine and Surgery, Bharati Vidyapeeth Medical College, Pune, IND; 3 Internal Medicine, California Institute of Behavorial Neurosciences and Psychology, Farfield, USA

**Keywords:** pancreatic cancer, inflammatory cytokines, depression, paraneoplastic syndrome, interleukin (il)-6

## Abstract

Pancreatic cancer is historically known for representing a challenge for both diagnosis and treatment. Despite the advances in medicine, science, and technology, it remains the third leading cause of cancer-related deaths in the United States. The association between pancreatic cancer and major depression preceding the diagnosis is well known; however, it is still poorly understood, being considered an obscure piece of the puzzle the disease represents. It has been characterized as a paraneoplastic syndrome caused by the dysregulation of inflammatory cytokines, especially interleukin-6 (IL-6). Despite many types of studies describing the association, researchers have been reluctant to recommend it as a screening tool or early marker of the disease, mainly because of the non-specific nature of depression and anxiety in the studied patients. In this literature review, we aim to better understand the relationship between pancreatic cancer and major depression and characterize the immunologic mechanism of action behind the association.

## Introduction and background

Pancreatic cancer is the third leading cause of cancer-related deaths in the United States, despite accounting for only 3.2% of all new cancer cases. It is estimated that in 2020, 57,600 Americans will be diagnosed with pancreatic cancer, and more than 47,050 will die from the disease. Adenocarcinoma, the most common type, accounts for about 90% of cases [1]. 

Pancreatic cancer has one of the highest mortality rates of all major cancers, with five-year relative survival for all stages combined of only 6%-10%. Even when diagnosed at its earliest stage, the five-year relative survival is 39.4%. However, at the time of diagnosis, 52% of cases represent advanced stages with distant metastases, while only 11% of cases are diagnosed at a localized stage [1]. This is attributed to the lack of specific symptoms exhibited by patients early in the disease. Certain risk factors for the development of pancreatic cancer have been identified, such as tobacco smoking, diabetes mellitus, obesity, age, and genetic factors, like inherited mutations in the breast cancer genes 1 and 2 (BRCA1 and BRCA2). 

The symptoms experienced by patients with pancreatic cancer are often non-specific, such as pain localized in the back or upper abdomen, nausea, vomiting, unintended weight loss, and jaundice. The incidence and nature of depression preceding the diagnosis of pancreatic cancer are less understood. Case-control studies have found that diagnosed patients were more likely to have experienced depression/sadness, fatigue, and difficulty concentrating in the year preceding the diagnosis [2]. However, the use of depression as an early marker for the condition is still controversial due to lack of current clinical evidence pointing to depression as a feasible screening tool [3]. 

Inflammation is believed to play a crucial role in depression experienced by pancreatic cancer patients. Both pancreatic cancer and major depressive disorder (MDD) have been associated with elevated levels of inflammatory cytokines, such as interleukin-6 (IL-6) [4,5]. Furthermore, cancer patients with depression have been found to have higher plasma levels of IL-6 in comparison to cancer patients without depression or healthy people [6]. Together, these findings could represent the mechanism described behind this commonly known but not yet fully understood association. In this review article, we aim to understand the pathophysiology behind cytokine-related depression in patients with pancreatic cancer.

## Review

Review Criteria 

We thoroughly searched PubMed Database in May 2020 for articles involving pancreatic cancer and depression, inflammatory cytokines and pancreatic cancer, and inflammatory cytokines and depression to identify the underlying mechanism of this widely known but poorly understood association. Articles were excluded if they focused on MDD after the diagnosis of pancreatic cancer as opposed to depression preceding the diagnosis, as well as studies of patients who had a long-standing history of psychiatric disease unrelated to the diagnosis of pancreatic cancer. 

After a thorough search and screening, we selected 28 articles; out of these, 12 articles focused on the relationship between pancreatic cancer and major depression, 12 articles focused on the association of IL-6 and inflammation with the development and progression of pancreatic cancer, and 6 articles focused on the association between IL-6 and major depression; some of them were included in more than one subcategory. A second search was conducted in June 2020 to look for recently published studies, with no new results. 

Discussion 

The relationship between pancreatic cancer and major depression has been the subject of interest of many researchers since Yaskin described it for the first time in 1931. He described the association with the triad of depression, anxiety, and “sense of impending doom” [7]. Ever since then, literature review pointing to depression as a precursor to a pancreatic cancer diagnosis has prompted studies to consider it as an early sign of the disease, many in the hope of using it as a testable marker, considering that currently there is no screening test for the disease. In this review article, we hope to better understand the correlation between depression and anxiety preceding a diagnosis of pancreatic cancer, and the pathophysiology and immunologic mechanisms that may explain the association. 

Pancreatic Cancer: Pathophysiology, Survival, and Diagnosis 

Pancreatic ductal adenocarcinoma is the predominant type of pancreatic cancer, accounting for approximately 90% of cases. Of these, about 90% are sporadic and 10% are familial or inherited cancer syndromes [8]. Despite the advances in science and technology, pancreatic cancer still has a grim prognosis, with a five-year survival rate of only 6%. Currently, surgery offers the best hope of survival. However, only 20% of patients are eligible for surgical resection at diagnosis, and as usually, the disease presents itself at an advanced stage [9]. Early detection has shown improved survival rates [10]. 

Pancreatic intraepithelial neoplasia (PanIN) is identified as the precursor lesion for pancreatic cancer, and it progresses through PanIN 2 (low-grade dysplasia) to PanIN 3 (high-grade dysplasia or carcinoma in situ) before becoming invasive. Invasive pancreatic cancer is classified by its size, and over 90% of them are diagnosed when they are relatively large (>2 cm or T2 according to tumor-node-metastases classification). Stage IV lesions are defined by having distant metastases and are not considered candidates for surgical resection [11]. For pancreatic cancer to progress from intraepithelial neoplasia to invasive disease, it needs to accumulate many genetic mutations. Telomere shortening and KRAS mutation have been described as early genetic changes, while SMAD4 and TP53 are considered late mutations in the disease progression [12]. 

Pancreatic cancer is usually diagnosed about two months after the onset of non-specific symptoms, and death occurs approximately six months after diagnosis. The use of an abdominal CT scan, a generally expensive test, as the first tool to diagnose the disease may partially explain the diagnostic delay. However, even when it is diagnosed at an early stage with a small tumor confined to the pancreas, the five-year survival is still only about 40% after surgical resection. This is often attributed to unknown metastatic disease at the time of diagnosis [12]. In addition to resection, radiation and chemotherapy have shown to slightly improve survival, with the latter showing an improvement from a median of three to six months to a median of six to nine months [13]. 

IL-6 and Cytokine Profile in Pancreatic Cancer 

**Figure 1:**
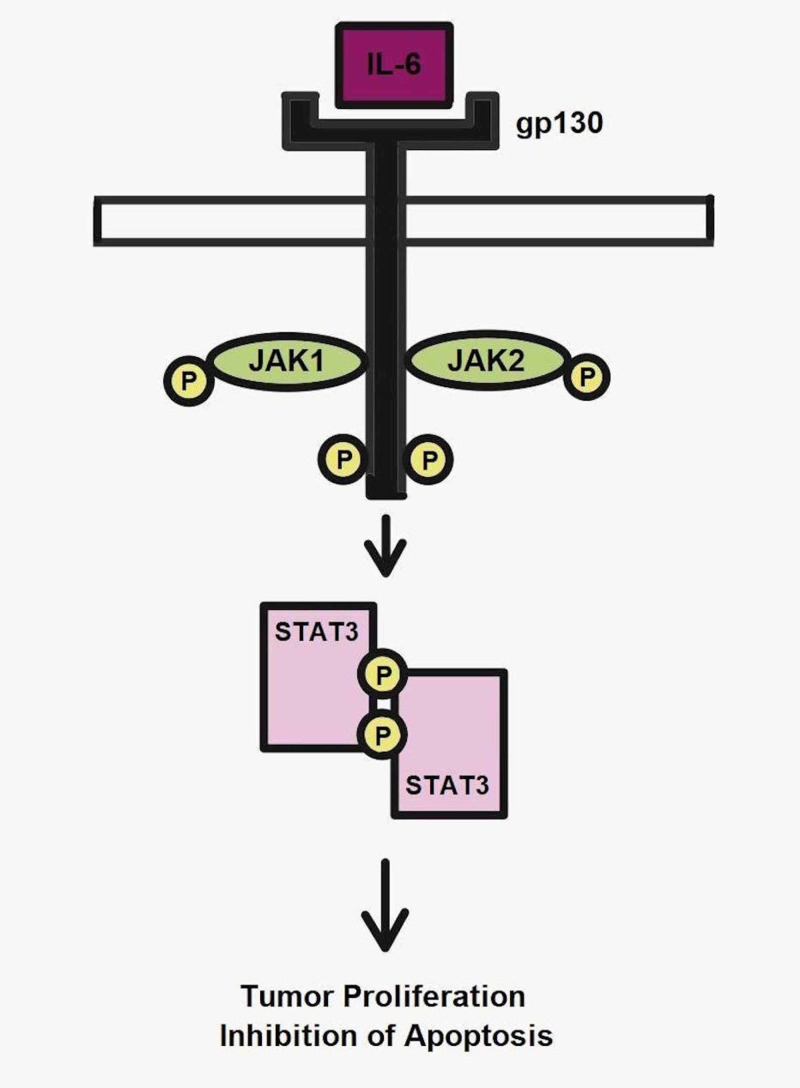
IL-6 and the JAK/STAT pathway in neoplasia. IL-6, interleukin-6; JAK/STAT, Janus kinase/signal transducer and activator of transcription

Inflammation has been recognized as one of the main features in the development and progression of cancer. The proinflammatory cytokine IL-6, normally produced by macrophages and monocytes, is significantly increased in pancreatic cancer cells compared to normal cells of a healthy pancreas [4,14]. IL-6 executes its function by binding to its receptor via the classic pathway or the trans-signaling pathway. Both of them lead to the activation of glycoprotein 130 (gp130) and its associated Janus kinase/signal transducer and activator of transcriptor proteins (JAK/STAT) pathway, leading to tumor growth and immunosuppression [15]. Therefore, elevated levels of IL-6 enhance the proliferation of tumor cells. Even further, they might promote an anti-apoptotic environment, rendering the cancer cells resistant to chemotherapy and immunotherapy [16]. Figure 1 shows the overall mechanism of action of IL-6 on the JAK/STAT activation pathway.

IL-6 has also been correlated to pancreatic cancer comorbidities, such as cachexia and depression [17]. This proinflammatory cytokine is thought to cross the blood-brain barrier in pancreatic cancer patients, as evidenced by its increased levels in both the blood and cerebrospinal fluid of study cases [5]. Furthermore, several studies have hypothesized IL-6 to be a prognostic indicator of survival and response to therapy, and a high IL-6 level usually indicating a poorer outcome [18]. Recent studies have explored IL-6 as a therapeutic target in pancreatic cancer; however, the available data are still limited [19]. 

Other inflammatory cytokines seem to play an important role in pancreatic cancer. Torres et al. described the serum cytokine profile of pancreatic cancer patients. They found significantly overexpressed levels of fibroblast growth factor 10 (FGF-10), keratinocyte growth factor 2 (KGF-2), chemokine ligand 11 (CXCL11), interferon-inducible T cell alpha chemokine (I-TAC), oncostatin M (OSM), glycoprotein nonmetastatic melanoma protein B, and stem cell factor (SCF). The cytokines CD30 ligand, tumor necrosis factor (TNF) superfamily member 8, chordin-like 2, FGF-10, KGF-2, I-TAC, CXCL11, OSM, and SCF were differentially expressed in response to treatment, rendering them as possible predictive biomarkers of response to therapy with gemcitabine and erlotinib [20]. 

Cytokine Profile in Major Depression and Its Association With Pancreatic Cancer 

Depression and psychosocial stress are well-known reactions to the diagnosis of cancer. However, it is increasingly being considered as related to the disease itself, especially preceding the diagnosis of pancreatic cancer. Inflammatory cytokines are known to affect monoaminergic and glutamatergic systems, which suggests a specific subtype of inflammatory cytokine-associated depression [21]. Many studies have described evidence that inflammatory cytokines may influence the brain and increase the risk of mood and psychiatric disorders. A meta-analysis of cytokines in major depression published by Dowlati et al., which excluded patients with any medical comorbidities, still found a strong association between depression and increased levels of IL-6 and TNF-ɑ [22]. 

Since the 1931 report by Yaskin, many researchers have noted the association of depression and anxiety preceding the diagnosis of pancreatic cancer, with one study reporting psychiatric symptoms preceding the medical diagnosis in between 33% to 45% of patients [7]. Similarly, Olson et al. described a case-control study that included pancreatic cancer patients from Memorial Sloan Kettering Cancer Center and healthy controls. They found that cases were more likely than controls to have experienced fatigue, difficulty concentrating, or depression/sadness in the year before diagnosis [2]. Several case series and case reports have also described a high incidence of depression before the diagnosis of pancreatic cancer; however, they were unable to identify a causal association [23,24]. 

The mechanism of the relationship between depression and pancreatic cancer is still poorly understood; however, one of the widely considered theories describes the increased levels of IL-6, among other inflammatory cytokines, as the link to major depression preceding the cancer diagnosis [25,26]. A study published by Breitbart et al., which compared patients with adenocarcinoma of the pancreas who did and did not have major depression and healthy participants, found an association with depression and IL-6, and significantly higher levels of IL-6 in pancreatic cancer patients [26]. A different study found that pancreatic cancer patients with depression had significantly increased levels of IL-6 than both healthy subjects and cancer patients without depression, further adding to this theory [27]. 

The depression and anxiety preceding pancreatic cancer diagnosis, therefore, could represent a paraneoplastic syndrome associated with the disease itself rather than a consequence of the diagnosis. However, the use of depression as a screening tool or early marker of the disease is still controversial, with many researchers doubting its utility as a marker of the condition [3]. 

## Conclusions

Even though pancreatic cancer remains one of the main causes of cancer-related deaths in the United States, its early diagnosis remains a challenge despite the advances in science and technology. Many aims at developing a cost-effective screening tool have failed, and the five-year survival remains low. This has prompted many researchers to find alternative clues to suspect the diagnosis, in the hope of it, leading to earlier detection and better prognosis. There is growing evidence documenting the association between pancreatic cancer and major depression preceding the diagnosis. So far, the available literature has described it as a possible paraneoplastic syndrome caused by the dysregulation of inflammatory cytokines, especially IL-6. However, the use of depression and anxiety as early markers or screening tools for the disease remains controversial. Combined with the high cost of diagnostic tests that would be able to identify the disease, this contributes to the reluctance to recommend the routine use of major depression as a screening tool. While the aim of this review is not to recommend the use of depression as a screening tool for this group of patients at the moment, we do consider it is important to have a better understanding of the pathophysiology and the mechanism of action underlying the association since it could eventually lead to the development of better screening tools, treatment targets, and ultimately, a more comprehensive care plan for our patients. We highly recommend continuing the studies of the disease and the economic support in the development of a better screening tool; as of now, depression and anxiety remain a relatively complex and not fully understood association that has not proved better outcomes in the early diagnosis of pancreatic cancer. 
